# Characterization of diverse Cas9 orthologs for genome and epigenome editing

**DOI:** 10.1073/pnas.2417674122

**Published:** 2025-03-12

**Authors:** Gabriel L. Butterfield, Dahlia Rohm, Avery Roberts, Matthew A. Nethery, Anthony J. Rizzo, Daniel J. Morone, Lisa Garnier, Nahid Iglesias, Rodolphe Barrangou, Charles A. Gersbach

**Affiliations:** ^a^Department of Biomedical Engineering, and Center for Advanced Genomic Technologies, Duke University, Durham, NC 27708; ^b^Department of Food, Bioprocessing and Nutrition Sciences, North Carolina State University, Raleigh, NC 27606

**Keywords:** CRISPR, genome editing, Cas9

## Abstract

CRISPR-Cas9 systems have revolutionized biotechnology, creating diverse new opportunities for biomedical research and therapeutic genome and epigenome editing. Despite the abundance of bacterial CRISPR-Cas9 systems, relatively few are effective in human cells, limiting the overall potential of CRISPR technology. To expand the CRISPR-Cas toolbox, we characterized a set of type II CRISPR-Cas9 systems from select bacterial genera and species encoding diverse Cas9s. Four systems demonstrated robust and specific gene repression in human cells when used as nuclease-null dCas9s fused with a KRAB domain and were also highly active nucleases in human cells. These systems have distinct protospacer adjacent motifs (PAMs), including AT-rich motifs and sgRNA features orthogonal to the commonly used *Staphylococcus aureus* and *Streptococcus pyogenes* Cas9s. Additionally, we assessed gene activation when fused with the p300 catalytic domain. Notably, *S. uberis* Cas9 performed competitively against benchmarks with promising repression, activation, nuclease, and base editing activity. This study expands the CRISPR-Cas9 repertoire, enabling effective genome and epigenome editing for diverse applications.

## Significance

CRISPR-based technologies have broadly enabled genome editing over the past decade, however, current approaches are disproportionately based on a single Cas9 effector with select efficiency and constrained targeting specificity. Here, we assess a diversity of Cas9 orthologs and describe four effectors that function in mammalian cells, with the *Streptococcus uberis* Cas9 as a particularly promising chassis for genome, transcriptome, and epigenome editing. This orthogonal effector is competitive against benchmarks and allows for complementary and flexible targeting of diverse genetic sequences for next-generation genome editing.

Since the initial discovery of CRISPR-Cas systems in bacteria (1), numerous orthologs have been characterized, spanning at least six types and 33 subtypes (2). Despite this diversity, type II systems, which utilize the single effector protein Cas9 to bind and cut DNA (3–5), remain the first choice for the majority of biotechnology and biomedical research applications. The ease of reprogramming Cas9 target sites has also ushered in a wave of potential genome editing therapeutics. For example, early in vivo gene editing efforts harnessed Cas9 nuclease activity to remove or induce skipping of mutated exons in disease models of Duchenne Muscular Dystrophy (6–8), and a phase III clinical trial is now underway for transthyretin amyloidosis (NCT06128629). A particularly exciting recent development is the approval of the first Cas9 therapeutic, Casgevy (9, 10), which disrupts an erythroid-specific *BCL11A* enhancer leading to upregulation of fetal hemoglobin as a treatment for sickle cell disease and beta thalassemia.

The discovery that nuclease-null Cas9 (dCas9) could function as a modular DNA targeting tool without inducing DNA breaks led to a simple and robust technology for epigenome editing, allowing programmable control of gene expression (11–15). Further advancements led to base editing (16, 17) and prime editing (18) technologies, enabling precise alteration of target DNA sequences. Mechanistically, Cas9 requires the presence of a specific PAM sequence adjacent to the target sequence, highlighting the need for additional diverse Cas9 orthologs with alternative PAM sequences and distinct sequence patterns to enable broad and comprehensive targeting. Furthermore, orthogonal tracrRNA sequences among different Cas9 orthologs allow for targeting of multiple Cas9s to unique sites within the same cell (19). Collectively, these advances underscore the vast potential of CRISPR-Cas9 technology in developing a variety of editing tools and potential therapeutics (20).

To address the need for additional Cas9 orthologs, we employed a bioinformatic approach to find uncharacterized type II CRISPR-Cas systems, focusing on bacterial genera that are enriched in Cas9 orthologs and broadly comprise diverse and nonpathogenic species (21–23) that presumably would not be targeted by human preexisting immunity. These Cas9 proteins were optimized for mammalian expression and initially tested as dCas9-KRAB fusions for gene repression in human cells. Several candidates, including those from *Streptococcus iniae*, *Streptococcus gallolyticus*, *Streptococcus lutetiensis*, *Streptococcus parasanguinis*, and *Streptococcus uberis*, showed significant repression, while *S. uberis* exhibited competitive nuclease and base editing activity against Cas9 benchmarks. Empirical PAM determination confirmed and expanded the targeting ranges of these systems, enabling broader scope and more precise genome-wide editing. These Cas9s were also effective in gene activation and at modulating therapeutically relevant targets, such as *PCSK9* repression, broadening the CRISPR-Cas9 repertoire for genome and epigenome editing.

## Results

### Bioinformatic Identification and Initial Characterization of Uncharacterized Cas9 Orthologs.

**Fig. 1.**
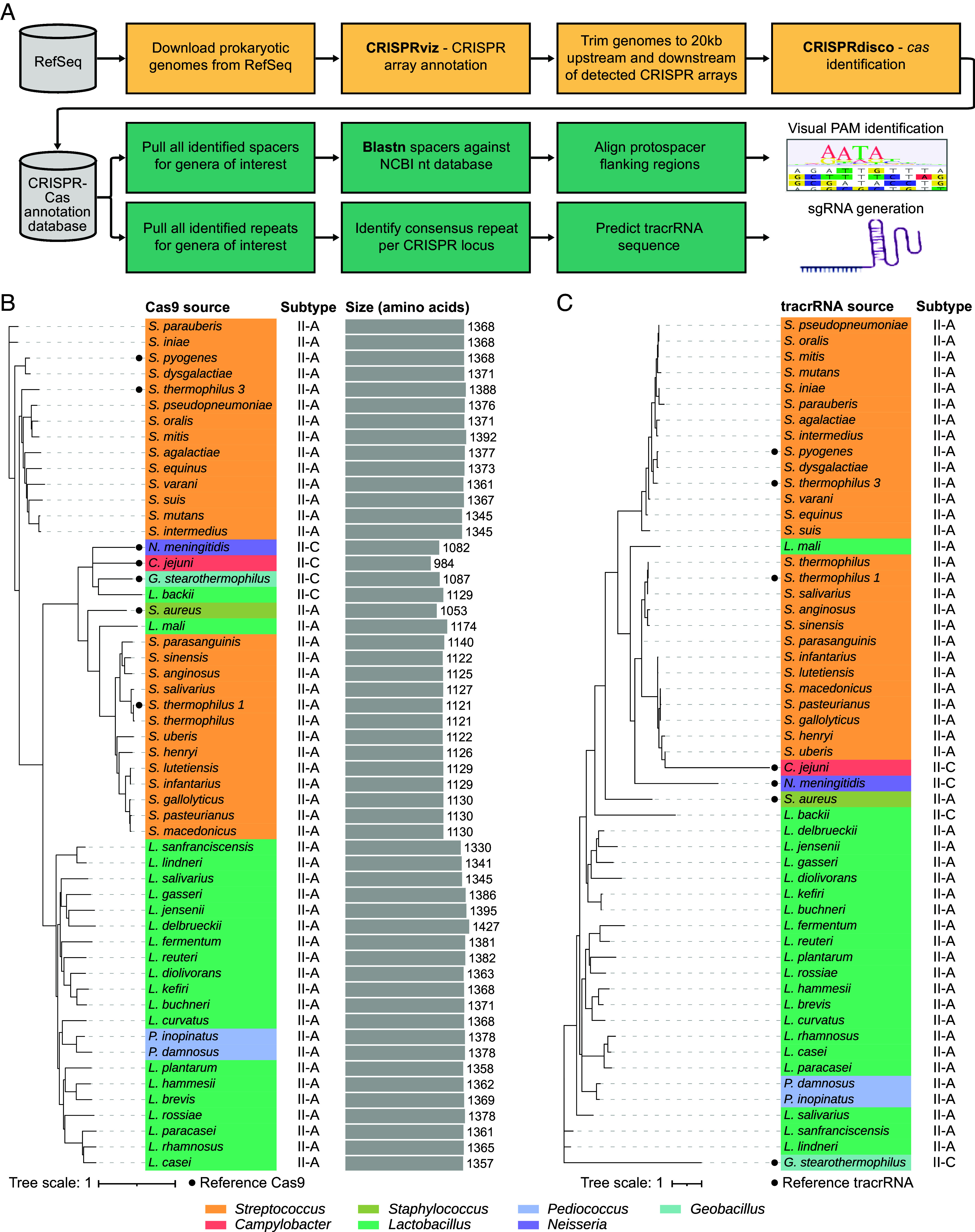
Identification and phylogenetic analysis of Cas9 orthologs and associated tracrRNAs. (*A*) Bioinformatic workflow depicting the mining of candidate Cas9 orthologs from RefSeq. (*B*) Phylogenetic tree of Cas9 protein candidates. The Cas9 source is colored according to genus and, for each Cas9, the associated CRISPR-Cas system subtype and amino acid length is shown. Label colors are based on the source genus and reference Cas9 proteins are included for comparison. (*C*) Phylogenetic tree of tracrRNAs associated with the Cas9 candidates in (*B*). Label colors are based on the source genus and reference tracrRNAs are included for comparison.

We used the CRISPRdisco pipeline (23) to mine bacterial genomes from select *Lactobacillales* genera that are commonly used in the food supply chain, widely associated with agricultural samples and hypothesized to be compatible with human exposure based on their broad use in food manufacturing and historical healthy occurrence in human microbiomes (24) (Fig. 1*A*). We also focused on streptococci and lactobacilli because these genetically and functionally diverse genera are enriched in CRISPR-Cas systems in general and Cas9-associated type II systems in particular. For each candidate Cas9, we predicted a PAM through spacer-protospacer matching and designed a single guide RNA (sgRNA) based on CRISPR repeat and tracrRNA predictions (25). The selected Cas9 candidates range from approximately 1,100 to 1,400 amino acids in length and exclusively belong to type II-A CRISPR-Cas systems, except for a single type II-C ortholog (Fig. 1*B*). Phylogenetic trees, constructed based on protein sequence similarity, reveal that our selected candidates cluster into three main groups, with two clusters predominantly composed of Cas9 from *Streptococcus* and one cluster exclusively featuring Cas9 from *Lactobacillus* and *Pediococcus*. Notable exceptions among our candidates include the type II-C Cas9 from *L. backii*, which closely resembles reference type II-C Cas9 orthologs, and a Cas9 from *L. mali* that is clustered with the smaller Cas9 orthologs derived from *Streptococcus*. Additionally, the alignment of putative tracrRNA sequences associated with these Cas9 proteins revealed similar phylogenetic relationships, underscoring the coevolution of Cas9s and their corresponding tracrRNAs (Fig. 1*C*). This comprehensive analysis yielded a list of candidate Cas9 orthologs, each paired with a corresponding putative PAM and sgRNA, therefore enabling their preliminary functional characterization as epigenome editors (Datasets S1 and S2 for sequences of all 47 Cas9s and sgRNA).

### Characterization of Mammalian-Adapted CRISPR-dCas9 Systems for Epigenetic Gene Repression.

**Fig. 2.**
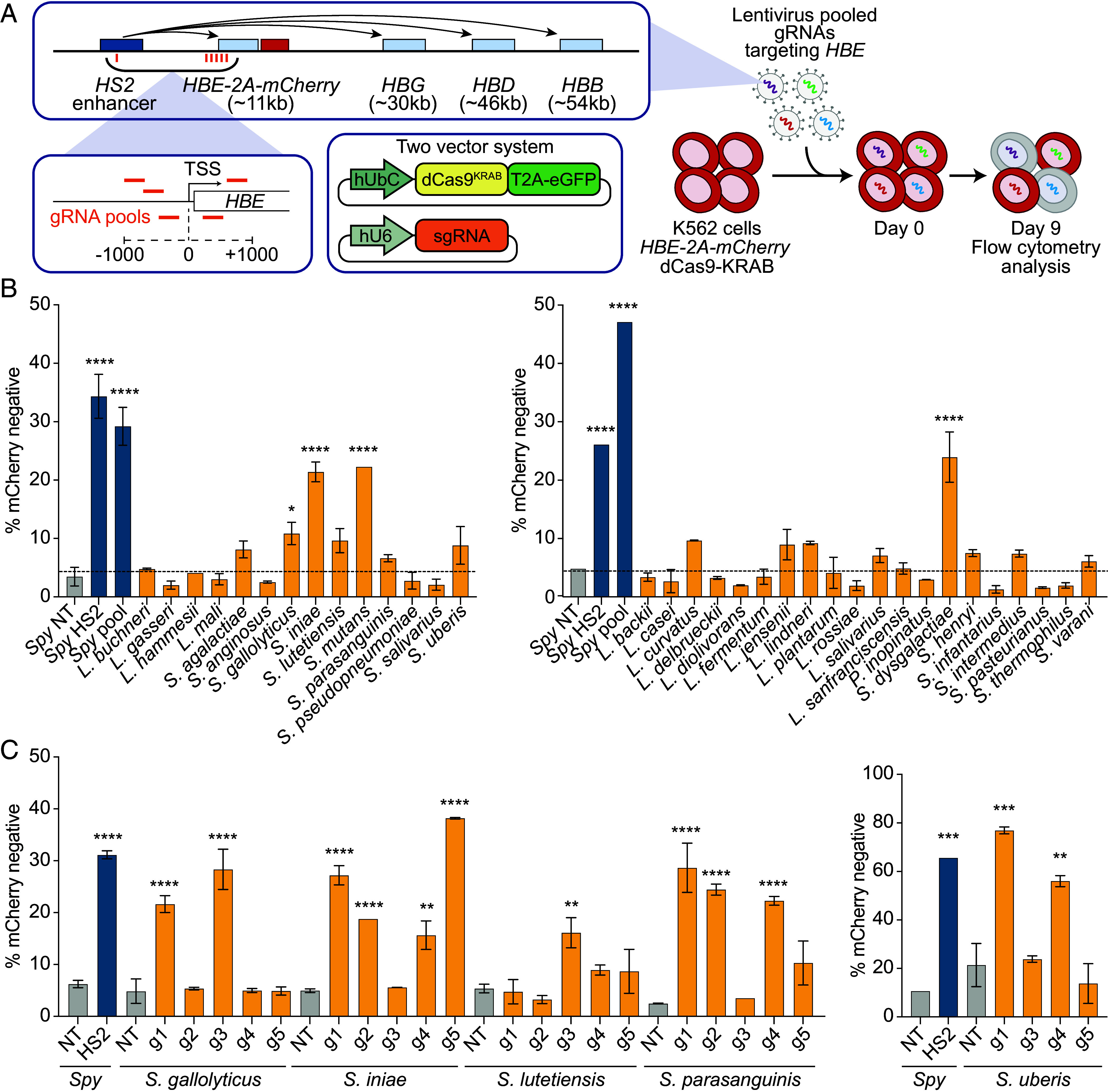
Repression of an endogenous *HBE*-*mCherry* reporter in human cells as an initial characterization of dCas9 activity. (*A*) Schematic of experimental design. A pool of 5 sgRNA targeting the promoter of *HBE* was designed for each dCas9. K562 cells containing an *HBE*-mCherry reporter were transduced with dCas9-KRAB-2A-EGFP followed by pooled lentivirus encoding the sgRNA. (*B*) Percent of cells in low *HBE* gate after transduction with dCas9-KRAB and pooled sgRNA lentivirus for all dCas9 systems. For some dCas9 systems, less than 1,000 dCas9-KRAB-EGFP positive cells were recovered and are not shown. Higher numbers indicate more effective repression. (*C*) Testing of individual sgRNAs from pools of sgRNAs for best-performing dCas9-KRAB fusions in (*B*). Data are shown as mean ± SD; n = 2 biological replicates. *P < 0.05, **P < 0.01, ***P < 0.001, ***P < 0.0001, one-way ANOVA followed by Dunnett’s multiple comparisons test.

The 47 wild-type Cas9 sequences were human codon-optimized and converted to nuclease-deactivated mutants (dCas9) via alanine substitutions at catalytic residues (3, 26) of the predicted RuvC and HNH nuclease domains (27, 28). These dCas9 sequences were fused with the ZNF10 KRAB repressor domain (29) and cloned into a lentiviral vector with an EGFP reporter to create a dCas9-KRAB-2A-EGFP lentiviral construct for epigenetic gene repression in human cells (Fig. 2*A*). We reasoned that while nuclease activity requires both DNA binding and cutting, transcriptional repression with a dCas9-KRAB fusion primarily relies on DNA binding, potentially expanding the number of functional Cas9 orthologs we could identify. To assess efficacy in repressing an endogenous gene in human cells, we used a K562 cell line containing the *HBE* gene tagged with an in-frame mCherry reporter protein, facilitating rapid evaluation of *HBE* knockdown (30) (Fig. 2*A*). Because repression efficiency is typically dependent on the target sequence, we designed up to five spacer sequences targeting the *HBE* promoter region for each computationally identified PAM sequence using ChopChop (31, 32) (Fig. 2*A* and Datasets S1 and S2). Considering the scarcity of some predicted PAM sequences, we searched for possible spacer sequences within a 1 kb region flanking the *HBE* transcription start site (Fig. 2*A*). We designed five spacers for each Cas9 except for *Pediococcus damnosus*, where the predicted PAM (NGATACA) was found only once (Datasets S1 and S2). As a control, we designed five spacers targeting the same TSS-flanking region for *Streptococcus pyogenes* Cas9 for the NGG PAM, using the same settings in ChopChop as for all other candidate Cas9 orthologs. A validated *S. pyogenes* sgRNA which targets the HBE enhancer and mediates potent downregulation of HBE was used as a positive control (30).

*HBE-mCherry* K562 cells were transduced with each dCas9-KRAB-2A-GFP construct and subsequently transduced with the corresponding pool of sgRNA lentiviruses (up to five sgRNAs per CRISPR system) (Fig. 2*A*). Nine days posttransduction, we assessed mCherry expression by flow cytometry. Out of 47 samples of dCas9-transduced cells, 34 exhibited a sufficient number of GFP-positive cells for analysis, indicating that low lentiviral expression of these uncharacterized Cas9 constructs is a common failure mode. Several dCas9-KRAB and sgRNA combinations markedly decreased *mCherry* expression (Fig. 2*B*). From those showing significant repression, we selected six dCas9s for further validation, prioritizing shorter Cas9 protein length, robust gene repression, and systems that have not otherwise been characterized. This validation process included individual testing of each of the five sgRNAs from the sgRNA pool used for *HBE-mCherry* repression. Notably, dCas9s from *S. iniae*, *S. gallolyticus*, *S. lutetiensis*, *S. parasanguinis*, and *S. uberis*, fused with KRAB, were successfully validated, demonstrating significant downregulation of *HBE-mCherry* with at least one sgRNA (Fig. 2*C* and *SI Appendix*, Fig. S1).

### Transcriptomic Analysis of Target Gene Expression.

**Fig. 3.**
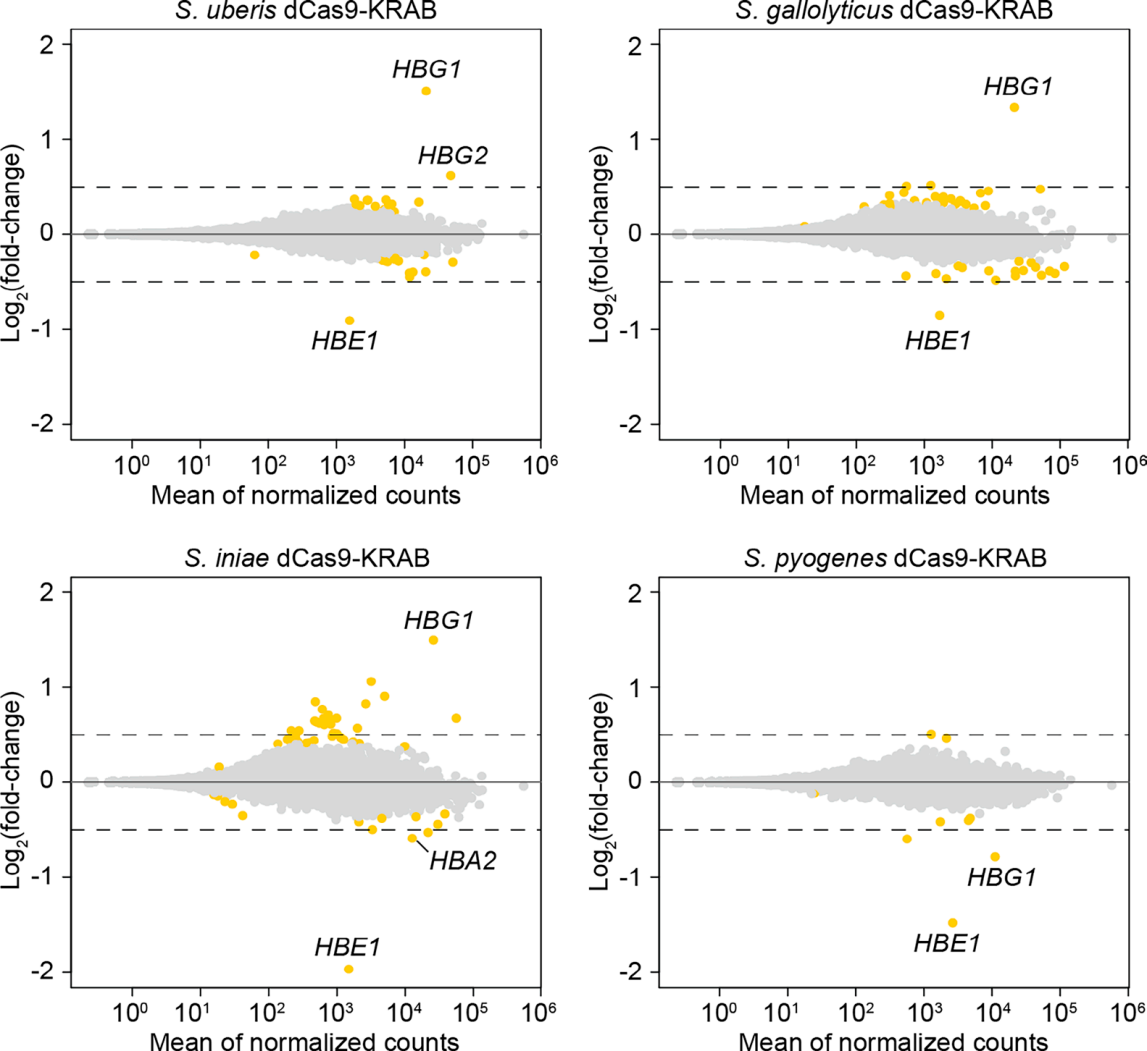
RNA-seq analysis of *HBE1* repression for dCas9-KRAB originating from *Streptococcus uberis, Streptococcus gallolyticus, Streptococcus iniae,* and *Streptococcus pyogenes* (control). Mean of normalized counts was plotted against log_2_(fold-change) using DEseq with shrunken residuals between *HBE1*-targeting and nontargeting sgRNA. *S. uberis, S. gallolyticus,* and *S. iniae* used the top promoter-targeting sgRNA, while *S. pyogenes* used an optimized and well-validated sgRNA targeting the HS2 enhancer. All genes differentially expressed between *HBE1*-targeting and NT sgRNA are shown in yellow regardless of fold change, with the dotted line indicating log_2_(fold-change) greater than 0.5 after shrinking of residuals. Each targeting and NT sgRNA was transduced and assayed in duplicate (*n* = 2 independent biological replicates per condition).

To evaluate the specificity of repression of the three dCas9-KRAB species with the strongest gene repression effects, we performed RNA sequencing analysis. We transduced K562 cells with lentivirus encoding dCas9-KRAB and a sgRNA targeting the *HBE* promoter and compared differentially expressed genes between samples transduced with a nontargeting sgRNA or the *HBE*-targeting sgRNA (Fig. 3). All three dCas9-KRAB proteins from *S. uberis*, *S. gallolyticus*, and *S. iniae* showed highly specific gene repression, with *HBE1* being the most significantly and strongly downregulated gene. All other downregulated genes with a log_2_(fold-change) greater than 0.5 were in the globin locus, such as *HBA2*. Modulation of additional globin genes is expected due to the known interdependency of gene expression at the globin locus (33–35). We used our previously validated HS2 enhancer-targeting *S. pyogenes* sgRNA (30) as a positive control, which showed similar levels of specific *HBE1* downregulation, as well as an expected downregulation of *HBG1* (Fig. 3). These differentially expressed genes were also validated by RT-qPCR, which confirmed significant changes in *HBE1* and *HBG1* in the expected direction (*SI Appendix*, Fig. S2).

### Comparative Structural Analysis and Empirical PAM Validation of Cas9 Orthologs.

**Fig. 4.**
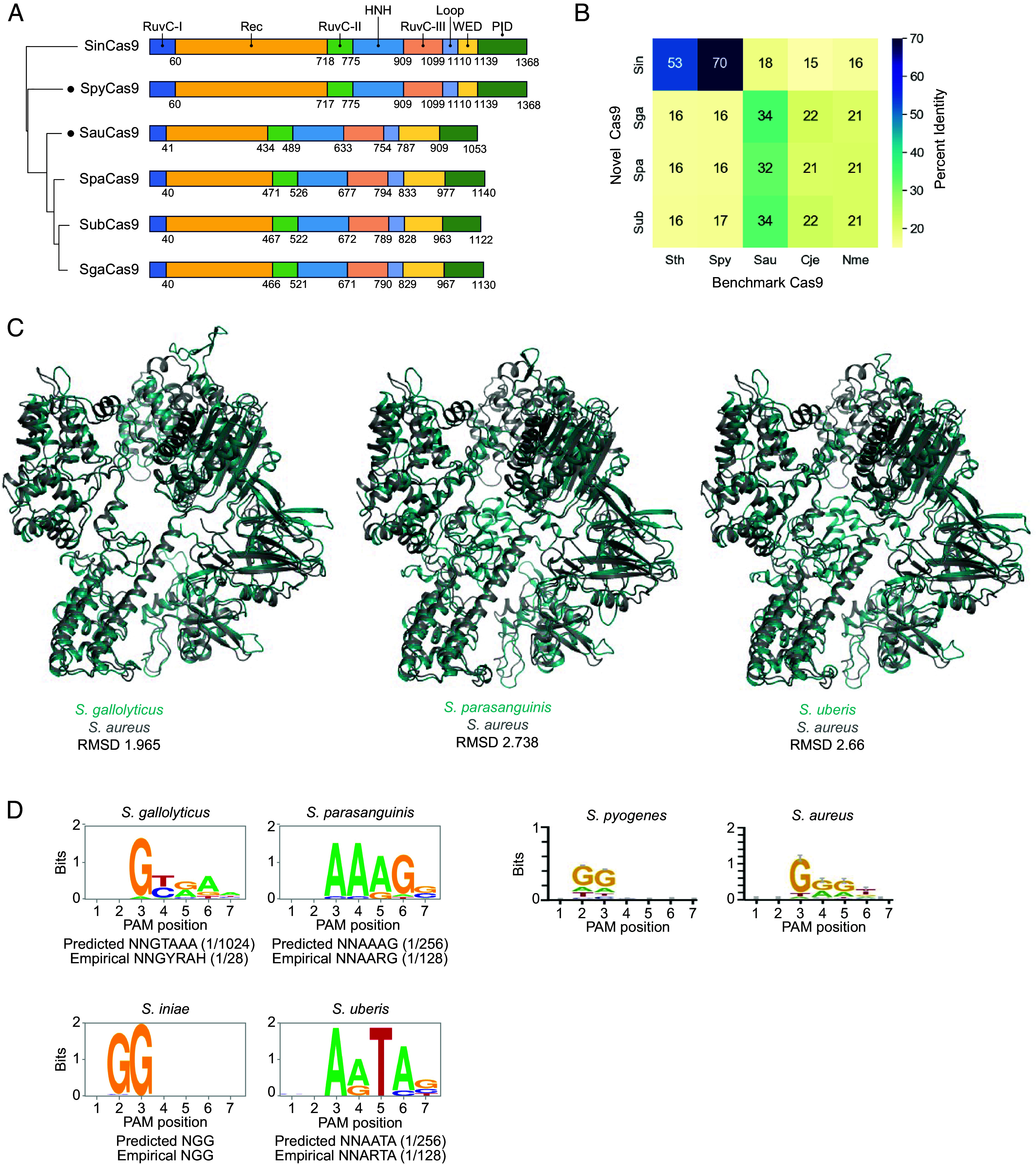
Cas9 structural comparison and PAM determination. (*A*) Phylogenetic tree of selected Cas9 orthologs. Sizes (amino acids) are indicated, with domain organization of each Cas9 based on alignment to *S. pyogenes* Cas9 (for *S. iniae*), or *Staphylococcus aureus* Cas9 (for *S. gallolyticus*, *S. parasanguinis*, and *S. uberis*). (*B*) Heatmap of percent amino acid sequence identity for pairwise alignment of uncharacterized Cas9s with previously characterized benchmark Cas9s. (*C*) Alignment of predicted structures for each Cas9 retrieved from the AlphaFold protein structure database. (*D*) Sequence logos for all empirically determined PAMs depleted at least 10-fold after in vitro cutting of a randomized PAM plasmid targeted by a constant spacer. In vitro transcription–translation reactions contained T7-driven Cas9 protein and sgRNA. PCR primers amplifying across the randomized PAM were used to generate amplicons for deep sequencing. Consensus PAMs for SpyCas9 and SauCas9 from Ran et al. (26) are shown on the right for comparison. SgaCas9: *S. gallolyticus;* SinCas9: *S. iniae;* SpaCas9: *S. parasanguinis*; SubCas9: *S. uberis;* Spy: *S. pyogenes.* Fraction of all possible DNA sequences that are targetable by each PAM is noted beside each predicted and empirical PAM.

We next set out to understand the relationship between Cas9 orthologs previously reported to be functional in mammalian cells and these Cas9 orthologs, narrowing our selection to four Cas9s based on their PAM flexibility and smaller size for potential AAV delivery. Sequence alignment between *S. iniae* (Sin), *S. gallolyticus* (Sga), *S. parasanguinis* (Spa), and *S. uberis* (Sub) Cas9s with those from *Staphylococcus aureus* (Sau) and *S. pyogenes* (Spy) highlighted distinct similarities and differences. SgaCas9, SpaCas9, and SubCas9 showed greater sequence identity to SauCas9 (32 to 35%), featuring a shorter REC domain and an extended wedge domain compared to SpyCas9 (Fig. 4 *A* and *B*). SgaCas9, SpaCas9, and SubCas9 also show 60 to 65% identity to each other. Conversely, SinCas9 demonstrated 70% sequence identity to SpyCas9, contrasting with an 18% identity to SauCas9 (Fig. 4*B*). Structural analyses, leveraging data from the AlphaFold database (36), revealed close structural alignment (1 to 3 Å RMSD) of SauCas9 with SgaCas9, SpaCas9, and SubCas9, despite sequence identities of only 32 to 34% (Fig. 4 *B* and *C*). Comparison to additional commonly used Cas9 proteins with evidence of activity in human cells, including those from *N. meningitidis* (Nme), *C. jejuni* (Cje), and *S. thermophilus* (Sth), revealed that SgaCas9, SpaCas9, and SubCas9 share 20 to 23% sequence identity with NmeCas9 and CjeCas9 (Fig. 4*B*) and a structural alignment RMSD of 4.7 to 9.7 Å (*SI Appendix*, Fig. S3). SgaCas9, SpaCas9, and SubCas9 share only 16% sequence identity (Fig. 4*B*) and a structural alignment RMSD of 11 to 21 Å with SthCas9 (*SI Appendix*, Fig. S3). Conversely, SinCas9 was more closely related to SthCas9, with 53% sequence identity, compared to only 15 to 16% identity to NmeCas9 and CjeCas9 (Fig. 4*B*). Comparison of predicted sgRNA structures reveals a similar grouping, in which *S. iniae* more closely resembles *S. pyogenes* with two 3’ hairpins. By contrast, sgRNAs from *S. gallolyticus*, *S. parasanguinis*, and *S. uberis* more closely resemble the *S. aureus* sgRNA, with a single 3’ hairpin, although differences between these orthologs are seen in the nexus and 3’ hairpin structure (*SI Appendix*, Fig. S4).

The predicted PAM sequence for the four validated Cas9 orthologs was further empirically verified using an in vitro cleavage assay with Cas9 proteins and sgRNAs targeting a constant spacer flanked by a randomized PAM of seven base pairs (7 N). This revealed distinct PAM preferences for each Cas9 after deep sequencing of cleaved amplicons, which consistently aligned with predicted PAM sequences (Fig. 4*D*). Notably, certain orthologs exhibited additional flexibility beyond the predicted PAMs. For example, while the predicted PAM sequence for SubCas9 was NNAATA, empirical determination revealed NNARTA, effectively doubling the potential target space.

To investigate the expanded target space provided by these orthogonal PAM sequences, we analyzed the theoretical base editing target space for each uncharacterized Cas9 protein, as well as SauCas9 and SpyCas9. We based our analysis on all single nucleotide variants (SNVs) that can be addressed by base editing (37). We found that the PAM sequences of our Cas9 proteins could potentially add 6,867 targetable sites to the 20,823 sites targetable by SpyCas9 and SauCas9 base editors, out of a total of 57,810 base-editable SNVs in ClinVar (*SI Appendix*, Fig. S5).

### *Streptococcus* Cas9 Orthologs Are Highly Active Nucleases in Mammalian Cells.

**Fig. 5.**
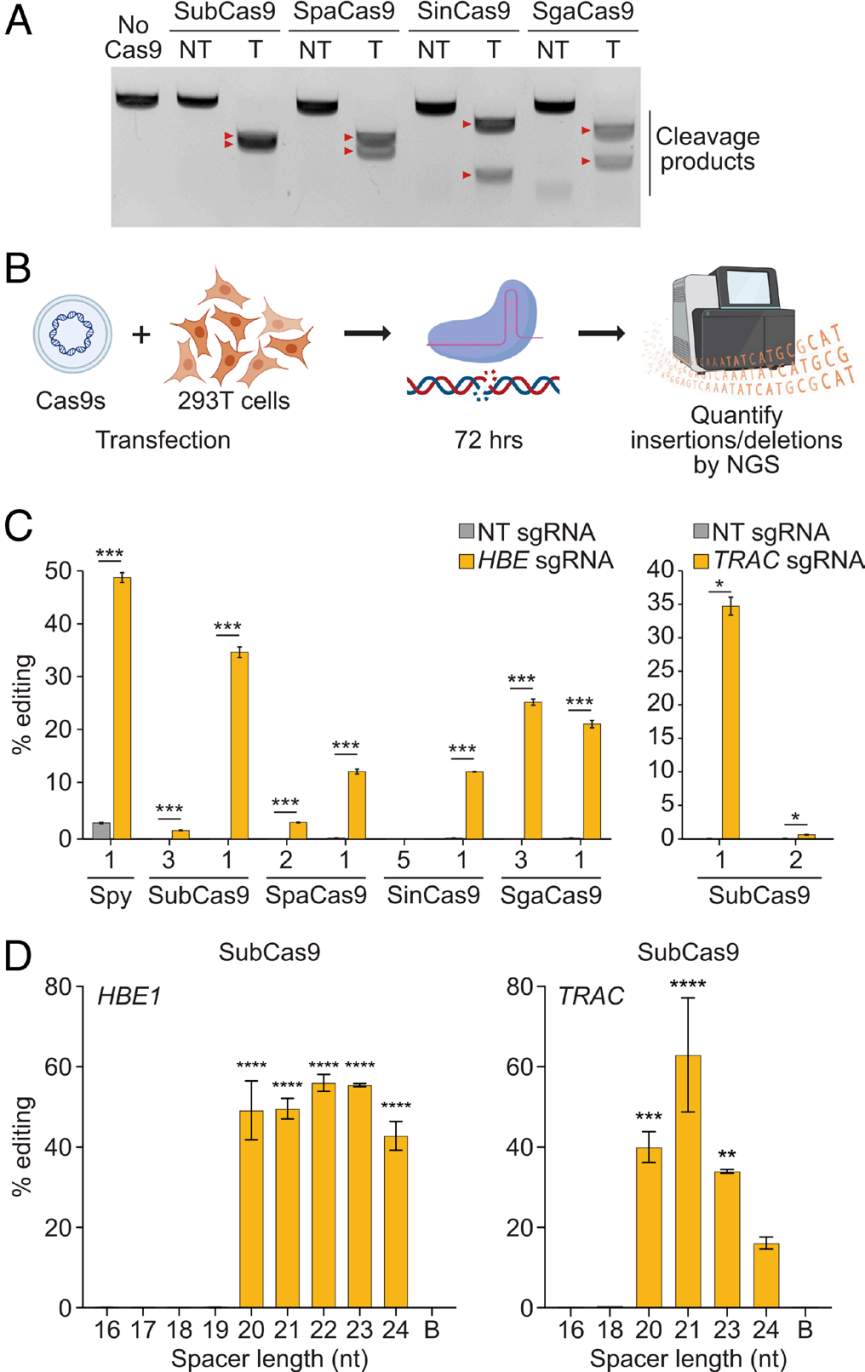
Nuclease activity in vitro and in mammalian cells. (*A*) In vitro cutting of a DNA amplicon from the *HBE* promoter by purified Cas9 protein and sgRNA for each Cas9. (*B*) Schematic representation of the genome editing activity experiments. (*C*) Genome editing activity of four *Streptococcus* Cas9s. Generation of insertions and deletions in the *HBE* promoter (*Left*) and *TRAC* coding sequence (*Right*) in HEK293T cells after cotransfection with plasmids encoding the respective Cas9 and corresponding sgRNA. Statistical significance was defined using student’s *t* test between targeting and nontargeting at each site, n = 3 biological replicates, ***P < 0.001. (*D*) Effect of protospacer length on the efficiency of SubCas9 editing. To assess the optimal protospacer length for SubCas9, single *HBE* (*Left*) and *TRAC* coding sequence (*Right*) sites were targeted by sgRNAs with protospacer lengths varying from 16 to 24 nt using cotransfection. “Background (B)” for each gene target is defined as indel-containing reads at the target site (*TRAC* or *HBE1*) in samples treated with the 20 bp-protospacer sgRNA for the opposite gene target (*HBE1* or *TRAC*). *n* = 2 independent biological replicates, **P < 0.01, ***P < 0.001, ****P < 0.0001; one-way ANOVA with Dunnett’s multiple comparison test. T: targeting sgRNA, NT: nontargeting sgRNA used as negative controls. Data plotted as mean ± SD.

To characterize the in vitro nuclease activity of these systems, each Cas9 protein was expressed in *Escherichia coli*, purified, and complexed with an in vitro transcribed sgRNA corresponding to the best *HBE*-targeting protospacer (Fig. 2*C*) for each Cas9. Incubating these ribonucleoprotein complexes (RNPs) with a DNA PCR amplicon containing the *HBE* target site demonstrated efficient in vitro nuclease activity in all cases (Fig. 5*A*). To further evaluate gene editing in human cells, plasmids encoding Cas9 proteins and sgRNAs were transfected into HEK293T cells (Fig. 5*B*). Deep sequencing of the endogenous *HBE* target sites in genomic DNA and quantification of indel frequencies demonstrated that all tested Cas9s are effective nucleases in mammalian cells (Fig. 5 *C* and *D*). SubCas9 exhibited the greatest cleavage activity, achieving ~35% editing at the best target, comparable to SpyCas9. SgaCas9 showed ~20% editing at both targets, while SpaCas9 and SinCas9 exhibited ~10% editing with at least one sgRNA. To demonstrate the versatility of SubCas9 in targeting various sequences, we designed two additional sgRNAs targeting the open reading frame of the *TRAC* gene, a therapeutically relevant target to prevent graft-versus-host disease in allogeneic T cell therapies (38), and transfected plasmids encoding Cas9 and these sgRNAs into HEK293T cells. Editing rates of ~35% were also observed for the best *TRAC*-targeting sgRNA, demonstrating that SubCas9 is a potent and robust nuclease in mammalian cells (Fig. 5*C*). Although the 20-nucleotide spacers used in these experiments proved sufficient for achieving editing, we sought to determine the optimal spacer length for the strongly active SubCas9 as the optimal length can vary between Cas9 systems (39, 40). We generated versions of the best *HBE* and *TRAC* sgRNAs for SubCas9 using spacer lengths ranging from 16 to 24 nucleotides (Fig. 5*D*). Spacer lengths below 20 nucleotides were found to completely ablate nuclease activity, while the optimal length was found to be 21 or 22 nucleotides, with the 21-nucleotide *TRAC* protospacer achieving 60% editing.

We also investigated the potential for *S. uberis* Cas9 to function as an adenine base editor (41, 42), utilizing this validated TRAC sgRNA. We generated a nickase Cas9 (nCas9) version of SubCas9, which creates a single-strand nick instead of a double-strand break in the target DNA and is thus suitable for base editing. *S. uberis* nCas9 was fused to the tadA adenine base editor domain and cotransfected into HEK293T cells along with TRAC or nontargeting sgRNA. We observed approximately 1% conversion of adenine to cytidine bases at protospacer positions 7, 9, 11, and 14 (*SI Appendix*, Fig. S6). These results confirm that *S. uberis* nCas9 can indeed function as a base editor and suggest an expanded editing window similar to that observed for SaCas9 base editors (43).

### Characterization of Mammalian-Adapted CRISPR-dCas9 Systems for Epigenetic Gene Activation.

**Fig. 6.**
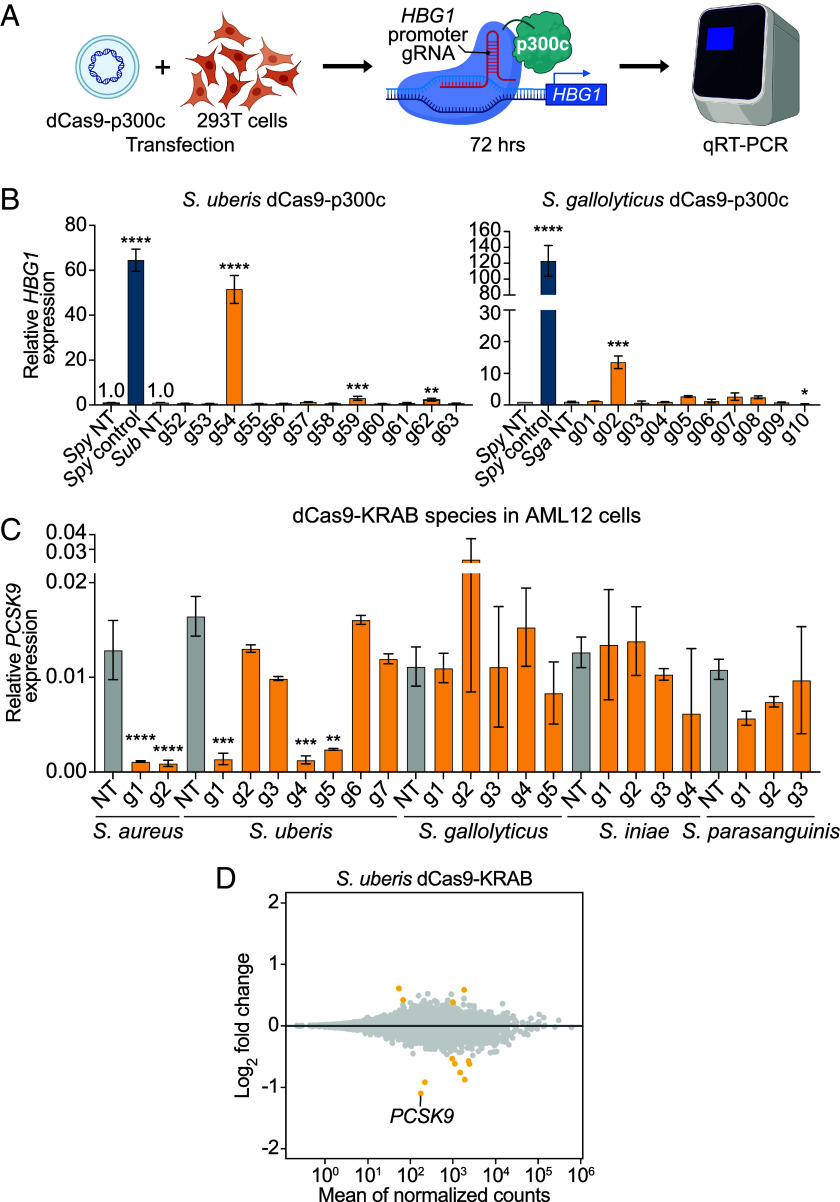
Epigenetic activation and repression at an additional target. (*A*) Schematic of dCas9-p300 gene activation experiment. HEK293T cells were transfected with plasmids encoding each dCas9-p300 and sgRNA. Seventy-two hours later, cells were harvested, and target gene expression was assessed by RT-qPCR. (*B*) RT-qPCR of *HBG1* expression in HEK293T cells expressing dCas9-p300 from *S. uberis* or *S. gallolyticus* dCas9 orthologs and corresponding sgRNAs targeting the *HBG1* promoter. (*C*) RT-qPCR of PCSK9 expression in AML12 cells after lentiviral transduction with dCas9-KRAB from each Cas9 ortholog and corresponding sgRNAs. *S. uberis* g1-4 have a 20 nt spacer, whereas g5-7 correspond to the same genomic locations as g1-3 (respectively), with a 21 bp spacer. For (*B*) and (*C*), *P < 0.05, **P < 0.01, ***P < 0.001, ****P < 0.0001. One-way ANOVA on ∆Ct values normalized to *GAPDH*, followed by Dunnett’s multiple comparison test relative to *S. pyogenes* dCas9-KRAB nontargeting control. multiple comparisons. *n* = 2 independent biological replicates. (*D*) RNA-seq analysis of *PCSK9* repression for *S. uberis* dCas9-KRAB. Mean of normalized counts was plotted against log_2_(fold-change) using DEseq with shrunken residuals between *PCSK9*-targeting and nontargeting sgRNA (n = 2). All genes differentially expressed between *PCSK9*-targeting and NT sgRNA are shown in yellow, regardless of fold change.

To further explore the epigenome editing capabilities of these dCas9 proteins, we fused each to the catalytic core of histone acetyltransferase p300, which we previously reported as a robust activator of promoters and enhancers (44). We then assessed *HBG1* gene activation in HEK293T cells after transfection with plasmids encoding dCas9-p300c and either an *HBG1* promoter-targeting sgRNA or nontargeting control (Fig. 6*A*). The dCas9-p300c fusions from *S. uberis* and *S. gallolyticus* successfully upregulated *HBG1*, while those from *S. iniae* and *S. parasanguinis* did not show any activation (Fig. 6*B* and *SI Appendix*, Fig. S7). Among the 12 sgRNAs tested with *S. uberis* dCas9-p300c, three significantly upregulated *HBG1* expression, with one achieving levels comparable to *S. pyogenes* dCas9-p300c paired with a previously optimized *HBG1* promoter-targeting sgRNA (Fig. 6*B*).

### Repression of a Therapeutically Relevant Target.

We selected *PCSK9* as an example of a therapeutically relevant target for gene repression, as *PCSK9* repression in hepatocytes leads to reduced cholesterol levels and is a promising clinical target for treatment of hereditary hypercholesterolemia (26, 45). Previous studies have demonstrated efficient *PCSK9* repression using *S. aureus* and *S. pyogenes* dCas9-KRAB systems both in vitro and in vivo (46, 47). For each dCas9 variant, we designed four to six sgRNAs targeting the region −200 to +300 bp relative to the *PCSK9* transcription start site, representing all experimentally determined PAM sequences. We included *S. aureus* dCas9-KRAB as a positive control. In the AML12 mouse hepatocyte cell line, *S. uberis* dCas9-KRAB significantly repressed *PCSK9* with two out of four tested sgRNAs, achieving repression levels comparable to *S. aureus* dCas9-KRAB using previously optimized sgRNAs (Fig. 6*C*). *S. parasanguinis* dCas9-KRAB achieved approximately 50% repression of *PCSK9*, while the remaining two dCas9-KRAB/sgRNA combinations did not significantly repress *PCSK9* expression (Fig. 6*C*). We further confirmed transcriptome-wide specificity of PCSK9 repression by *S. uberis* dCas9-KRAB using RNA sequencing analysis. After transduction of AML12 cells with *S. uberis* dCas9-KRAB and PCSK9-targeting or nontargeting sgRNA, the most significantly repressed gene was PCSK9 (Fig. 6*D*). Eight other transcripts were also significantly downregulated; however, these genes were also downregulated when PCSK9 is targeted by SauCas9 in AML12 cells (*SI Appendix*, Fig. S8), suggesting that these are downstream consequences of PCSK9 repression rather than off-target activity of *S. uberis* dCas9-KRAB.

## Discussion

Our initial characterization of 47 dCas9 effectors identified five orthologs with efficient gene repression activity in human K562 cells when fused to a KRAB domain. Further investigation of dCas9-KRAB variants from *S. uberis*, *S. gallolyticus*, and *S. iniae* revealed highly specific *HBE1* repression, as demonstrated by RNA-seq analysis. These Cas9s orthologs also showed potent nuclease activity in HEK293T cells, with *S. uberis* demonstrating particularly robust editing rates of up to 60%.

The versatility of these CRISPR systems was further demonstrated through gene activation experiments. dCas9-p300c fusions from *S. uberis* and *S. gallolyticus* successfully activated the *HBG1* gene when targeted to its promoter in HEK293T cells. Moreover, we observed significant repression of the therapeutically relevant *PCSK9* gene (26, 45) in the AML12 mouse hepatocyte cell line using dCas9-KRAB from *S. uberis* and *S. parasanguinis.* Thus, these four uncharacterized Cas9s orthologs substantially expand the limited repertoire of CRISPR-Cas9 systems known to function in mammalian cells. Their robust performance across multiple mammalian cell types, species, target genes, delivery methods, and functional modalities (repressors, activators, nucleases, and base editors) indicates that they are as versatile as the commonly used SpyCas9 and underscores their potential as versatile tools for gene regulation studies and therapeutic applications. Furthermore, the ability of *S. uberis* nCas9 to function as an adenine base editor expands its application range to include precise nucleotide modifications. Efficiencies of editing could likely be further improved in the future by additional optimization of fusion protein architectures specifically in the context of SubCas9.

Although our study initially included CRISPR-Cas9 systems from *Lactobacillus* (21), *Streptococcus* (24), and *Pediococcus* (2) species, all four high-performing Cas9s orthologs were derived from *Streptococcus* species, suggesting that this genus may be a rich source of CRISPR effectors suitable for mammalian cell applications. Our structural analyses using AlphaFold revealed that despite low sequence identity (~30%), these three Cas9s orthologs maintain high structural similarity to established systems like SauCas9, with RMSD values of 1–3 Å). This structural conservation hints at common features that likely contribute to their effectiveness in human cells.

The compact size of Cas9 proteins from *S. uberis* (1,122 aa), *S. gallolyticus* (1,130 aa), and *S*. *parasanguinis* (1,140 aa) makes them suitable for codelivery with sgRNA expression cassettes in single adeno-associated virus (AAV) vectors. Currently, the most widely used Cas9 ortholog for single AAV delivery is derived from *S. aureus*, a notable human pathogen. This frequent human exposure has led to the detection of antibodies and T cells targeting these Cas9 proteins in blood samples from healthy human donors (48–50). Moreover, prior immunization of naive mice with Cas9 led to immune-mediated elimination of Cas9-expressing cells following AAV gene therapy vector delivery (51). Thus, additional Cas9 orthologs may become important for circumventing anti-Cas9 immune responses. While the bacterial species we selected are typically nonpathogenic and commonly found in food or agricultural environments, which may reduce the likelihood of preexisting immunity in humans, this does not guarantee a lack of immunogenicity per se. Future studies could address this question by examining T cell responses to these Cas9 orthologs using peripheral blood mononuclear cells from a diverse set of human donors, or by conducting in vivo studies in humanized mouse models.

Importantly, the A/T-rich PAMs of SpaCas9 and SubCas9 complement the G/C-rich PAMs of established Cas9 variants (e.g., NGG for SpyCas9), expanding the range of target sequences for precise genome editing techniques such as base and prime editing. This increased flexibility in target site selection is crucial for overcoming current limitations in sgRNA design for precision gene therapies. Furthermore, their orthogonal sgRNA structures likely enable multiplexed deployment of CRISPR effectors (22). In conclusion, our identification and characterization of four uncharacterized CRISPR-Cas9 systems from *S. uberi*s, *S. gallolyticus*, *S*. *parasanguinis, and S. iniae* significantly enhance the CRISPR toolbox. These effectors open up possibilities for next-generation genome editing, offering improved targeting capabilities, potentially reduced immunogenicity, and versatile applications in both research and therapeutic contexts.

## Materials and Methods

### Cas9 Identification.

All available prokaryotic genomes were downloaded from the National Center for Biotechnology Information’s Reference Sequence Database (52) (May 2018). CRISPR arrays were identified using the CRISPR Visualizer software package (53). To facilitate more rapid processing of each genome, only the regions 20 kb upstream and downstream of identified CRISPR loci were kept for subsequent processing (genome trimming). Cas genes were annotated using the CRISPRdisco software pipeline (23). The resulting repeat, spacer, and *cas* sequences were stored in a Postgres database.

### PAM and tracrRNA Prediction.

To determine spacer identity, all spacer sequences for the genera *Lactobacillus*, *Pediococcus*, and *Streptococcus* were compared against NCBI’s Nucleotide Collection database (nt) via nucleotide BLAST (54) using default parameters with the following exceptions: -e-value 1e-3, -task blastn-short, -dust no. BLAST alignment matches that were viral or plasmid in origin were aligned, and PAMs were determined after visual inspection of the alignments in Geneious (55). A consensus repeat sequence for each CRISPR locus was determined by multiple alignment of all available repeat sequences per array. The consensus repeat was used to predict the putative tracrRNA sequence through the identification of the antirepeat sequence, as previously described (56). sgRNA sequences were generated by joining the repeat and predicted tracrRNA sequences to form a crRNA:tracrRNA duplex with a short nucleotide tetraloop: “CGAA,” as previously described (25).

### Cas9 and tracrRNA Phylogenetic Analyses and dCas9 Generation.

The Cas9 protein sequences were aligned using MUSCLE 3.8.425 with default parameters in Geneious (57). A consensus tree was generated using the Geneious Tree Builder in Geneious with a Jukes-Cantor model, a neighbor-joining tree build method, and 100 replicates. The tracrRNA sequences were aligned using MUSCLE with default parameters in Geneious. A tree was generated with Geneious by using the RAxML plugin with a GTR GAMMA nucleotide model, a rapid hill-climbing algorithm, and 20 replicates (58). Trees were visualized using iTOL (59). After aligning the protein sequences, each Cas9 ortholog was modified into a dead Cas9 (dCas9) by introducing specific single amino acid changes within the HNH and RuvC catalytic domains. These changes are analogous to the D10A and H840A mutations found in SpyCas9, which are known to deactivate the nucleolytic activity of the RuvC and HNH domains, respectively (3).

### Cas9 and sgRNA Cloning.

To generate dCas9^KRAB^ plasmids for all candidate dCas9s, we modified a lentiviral expression plasmid (Addgene no. 83925) by removing the U6 promoter and gRNA scaffolds and inserting two AgeI restriction sites separated by a short stuffer sequence, an SV40 nuclear localization sequence, and KRAB-2A between the promoter and GFP-2A-BlastR sequences. The sequences of the candidate Cas9s were supplied to Twist Biosciences for human codon optimization and homology-based cloning into the AgeI site in our custom expression plasmid.

Individual or pooled gRNA plasmids were cloned using Gibson assembly downstream of the hU6 promoter into a plasmid expressing a puromycin resistance gene from a separate, hUbC promoter. gRNA scaffolds and protospacers were ordered as long oligonucleotides (IDT) and extended via PCR (Q5, NEB).

For protein production, *E. coli* codon-optimized genes encoding nuclease-active Cas9 from *S. uberis, S. gallotyticus, S. parasanguinis, S. iniae*, and *S. pyogenes* were cloned into pET29B between the NcoI and PacI sites by Gibson assembly.

For plasmid used in nuclease experiments in HEK293T cells, nuclease active sites were restored in the human codon-optimized DNA encoding Cas9 from *S. ub., S. ga., S. pa., S. in.,* and *S. py.* and cloned between the NcoI and PmeI sites of Addgene plasmid 61357 by Gibson assembly to generate CMV-driven constructs optimal for transfection of mammalian cells.

### Lentiviral Transduction of K562 Cells.

K562 *HBE-mCherry* cells (30) were maintained in RPMI 1640 and used to test gene repression activity of each dCas9-KRAB with sgRNAs targeting the *HBE* promoter. K562 *HBE-mCherry* (40,000 cells) were transduced with dCas9-KRAB lentivirus (50µL) corresponding to each Cas9 in 500µL RPMI with 5 µg/mL polybrene. Three days later, the resulting dCas9-KRAB cell line was further transduced with pooled (Fig. 2*B*) or individual (Fig. 2*C*) sgRNA lentivirus (sgRNAs in a cassette containing a puromycin resistance gene) and selected with puromycin for 72 h. Then, 9 or 10 d posttransduction, cells were harvested and EGFP-positive cells were analyzed for mCherry repression on a flow Sony SH800 cytometer. For RNA-seq experiments, EGFP-positive cells were first sorted and collected, followed by sgRNA transduction, culture, and RNA extraction using the Qiagen RNeasy kit according to the manufacturer’s instructions.

### In Vitro PAM Determination.

The PAM sequence for each Cas9 protein was determined empirically as previously described (60). Individual 12 µL TXTL reactions were assembled consisting of 9.375 µL myTXTL Linear DNA Master Mix (Daicel Arbor Biosciences), 0.5 mM IPTG, 0.2 nM pTXTL-P70a-T7rnap (Daicel Arbor Biosciences), 2 nM Cas9 linear DNA containing a T7 promoter and 2 nM linear sgRNA expression gBlock, and 0.5 nM 7 N plasmid library. TXTL reactions were incubated at 29 °C for 16 h. The DNA was purified from the TXTL reactions with the Monarch PCR & DNA Cleanup Kit (NEB). DNA libraries were then amplified by PCR and subjected to Illumina sequencing to determine counts of each sequence.

### In Silico Analysis of Base Editing Targets.

All SNVs from ClinVar which are addressable by base editing (37) were analyzed for the presence of a PAM for each Cas9 which would place the variant within the editing window. For SpyCas9, the editing window used was protospacer positions 4 to 8. Protospacer positions 3 to 11 were considered to be the editing window for SauCas9 (43), as well as for each of SpaCas9, SgaCas9, and SubCas9. This window was used based on both the high degree of structural homology of SpaCas9, SgaCas9, and SubCas9 with SauCas9, as well as our experimental verification of the editing window with an SubCas9 adenine base editor (*SI Appendix*, Fig. S6).

### Protein Purification.

BL21 *E. coli* cells (Millipore EMD; MilliporeSigma, Burlington, MA) were transformed with Cas9 expression plasmid and plated on plates containing carbenicillin. Liquid cultures were then inoculated and allowed to grow at 37 °C until the OD600 was 0.6 to 0.8, and then induced with 0.5 mM IPTG and grown overnight at 18 °C. Cultures were then pelleted by centrifugation (10 min at 4,000×*g*). Cells were resuspended in lysis buffer, lysed by sonication, and spun at 20,000×*g* to remove cell debris. The lysate was then flowed over a 2 mL bed volume of Ni-NTA agarose (Qiagen), washed twice with wash buffer, and then once with wash buffer without triton. Protein was eluted by the addition of 10 mL elution buffer. Concentration was determined by qubit. The buffers used for protein purification included the following: Lysis Buffer (20 mM Tris-HCl pH 8.0, 500 mM NaCl, 10 mM imidazole, 5% glycerol, 1 mg/mL lysozyme, 1 tablet Complete protease inhibitor, EDTA-free); Wash Buffer (20 mM Tris-HCl pH 8.0, 500 mM NaCl, 20 mM imidazole, 0.5% triton x-100); Elution Buffer (20 mM Tris-HCl pH 8.0, 500 mM NaCl, 500 mM imidazole).

### In Vitro Transcription of sgRNA.

For in vitro cleavage reactions, sgRNA were produced by in vitro transcription using the Megashortscript kit (ThermoFisher, Waltham, MA) according to the manufacturer’s instruction for each sgRNA.

### In Vitro Nuclease Activity.

In a 50 µL reaction, 100 nM Cas9 protein and 100 nM sgRNA were combined with 6 nM target DNA in 150 mM NaCl, 20 mM tris pH 8, and 10 mM MgCl_2_. Reactions were incubated at 37 °C for two hours and then terminated by the addition of proteinase K (500 µg/mL final concentration) and RNase A (10 µg/mL) followed by a 10-minute incubation at 37 °C. DNA was then purified using Ampure XP DNA cleanup beads and analyzed on a 1% agarose gel.

### Nuclease Activity and Base Editing in HEK293T Cells.

HEK293T cells were maintained in DMEM supplemented with 10% FBS and 1x penicillin/streptomycin. The day before transfection, 200,000 HEK293T cells were plated in 24-well plates. Plasmids encoding nuclease-active Cas9 (200 ng) and sgRNA (300 ng) were transfected into HEK293T cells using lipofectamine 3000 according to the manufacturer’s instructions. For base editing, plasmids encoding *S. uberis* TadA-nCas9 (300 ng) and sgRNA (200 ng) were used. The *S. uberis* TadA-nCas9 plasmid was based on ABE8e (42). After 72 h, gDNA was extracted from transfected cells. Extracted gDNA was then subjected to PCR to generate amplicons flanking each target site and sequenced on an Illumina Miseq. The editing efficiency after cotransfection with SubCas9 and sgRNA was measured by next-generation sequencing (NGS). Custom python scripts were used to assess the number of reads containing insertions and deletions or direct A-to-G conversions in the case of base editing, relative to the wild type sequence at each target site.

### sgRNA Design for Activation and Repression.

sgRNAs for all activation and repression assays were designed using ChopChop version 3 (31, 32, 61) with the following settings differing from default: Custom PAM, G20 efficiency score, no repair profile predictions, no 5’ requirements for sgRNA, and custom sgRNA backbone. Sequences targeted the gene promoter and TSS-proximal regions, but searches were sometimes expanded to within 1,000 bp of the TSS depending on PAM frequency. See Dataset S1 for all sgRNA sequences, and Dataset S2 for sequences for all 47 Cas9s.

### *PCSK9* Repression in AML12 Cells.

AML12 cells were obtained from ATCC (cat no.CRL-2254) and cultured in DMEM/F12 (Thermo cat no.11320033) with 10% FBS, ITS-G (Gibco 41400045), and 40 ng/mL dexamethasone. To generate stable polyclonal dCas9-KRAB cell lines, AML-12 cells were transduced with lentivirus encoding dCas9-KRAB-2A-GFP-2A-BlastR and selected for 3 d with 3 µg/mL blasticidin. After antibiotic selection, GFP-expressing cells were isolated via cell sorting (Sony SH800Z in semipurity mode). To assay *PSCK9* repression in each dCas9-KRAB cell line with sgRNAs, cells were plated at 1.82E4 cells/cm^2^ and immediately transduced with sgRNA virus. Approximately 30 to 48 h after transduction, puromycin was added to fresh media at 1 µg/mL. Cells were selected with puromycin for 4 d. Cells were harvested with Trypsin 0.05% (Gibco cat no. 25300054) 9 d after transduction. RNA was extracted from frozen cell pellets with the Norgen Total RNA Purification Plus kit (Norgen cat no. 48300). Then, 500 ng RNA per sample was reverse-transcribed using the Superscript VILO cDNA synthesis kit (Invitrogen cat no. 11754050) and analyzed by RT-qPCR.

### RNA-seq Analysis.

K562 or AML12 cells were transduced as described above with vectors encoding dCas9-KRAB and appropriate sgRNAs. Cells (1 to 2 million) were pelleted and RNA was extracted using the RNeasy kit according to the manufacturer’s instructions. Standard RNA sequencing with polyA selection and ribosomal RNA depletion was performed by Azenta. Raw reads were processed into counts for each gene using the ENCODE standard RNA sequencing pipeline (62). Counts were then compared between *HBE*-targeting or PCSK9-targeting and nontargeting sgRNA samples using DEseq2 with independent filtering off and shrunken residuals.

## Supplementary Material

Appendix 01 (PDF)

Dataset S01 (XLSX)

Dataset S02 (XLSX)

## Data Availability

RNA seq data have been deposited in NCBI (PRJNA1137952) (63). All study data are included in the article and/or supporting information. Plasmids have been deposited with Addgene (Plasmids 227005–227012) (64–71).
