# Supplementary material for: Characterization of diverse Cas9 orthologs for genome and epigenome editing
Source: Proc Natl Acad Sci U S A. 2025 Mar 12;122(11):e2417674122. doi: 10.1073/pnas.2417674122 (PMC11929499; doi:10.1073/pnas.2417674122)
Supplement: Supplementary file 1 — Appendix 01 (PDF) [file pnas.2417674122.sapp.pdf]

**Supplemental Figures**

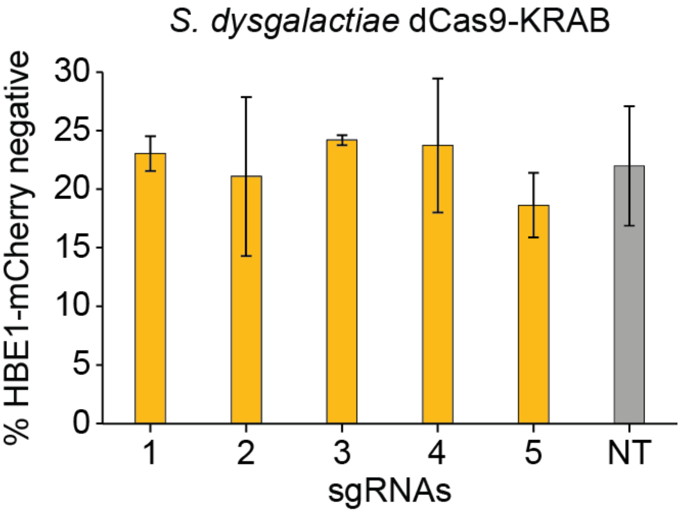


**Supplemental Figure 1.** *S. dysgalactiae* does not mediate *HBE1* repression with individual sgRNAs. K562 cells bearing mCherry tagged *HBE1* gene were transduced with lentivirus encoding *S. dysgalactiae* dCas9-KRAB followed by the indicated sgRNA. HBE-mCherry fluorescence was assessed by flow cytometry 10 days after transduction with sgRNA.

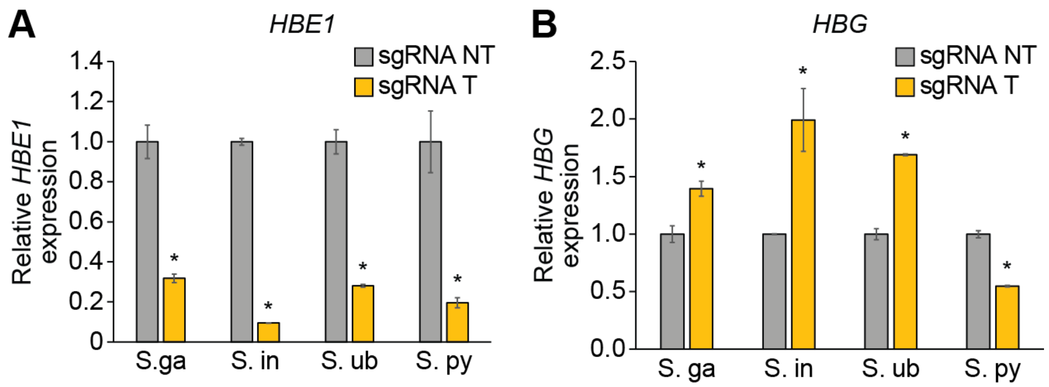


**Supplemental Figure 2**. RT-qPCR validation of differentially expressed genes identified by RNA sequencing. **A**. RT-qPCR analysis of *HBE1* expression in K562 cells following lentiviral transduction with dCas9-KRAB from each Cas9 ortholog and corresponding sgRNAs. **B**. RT-qPCR analysis of *HBG* expression in K562 cells under the same conditions as in (A). * $p < 0.05$, student's t-test comparing targeting to non-targeting sgRNA.

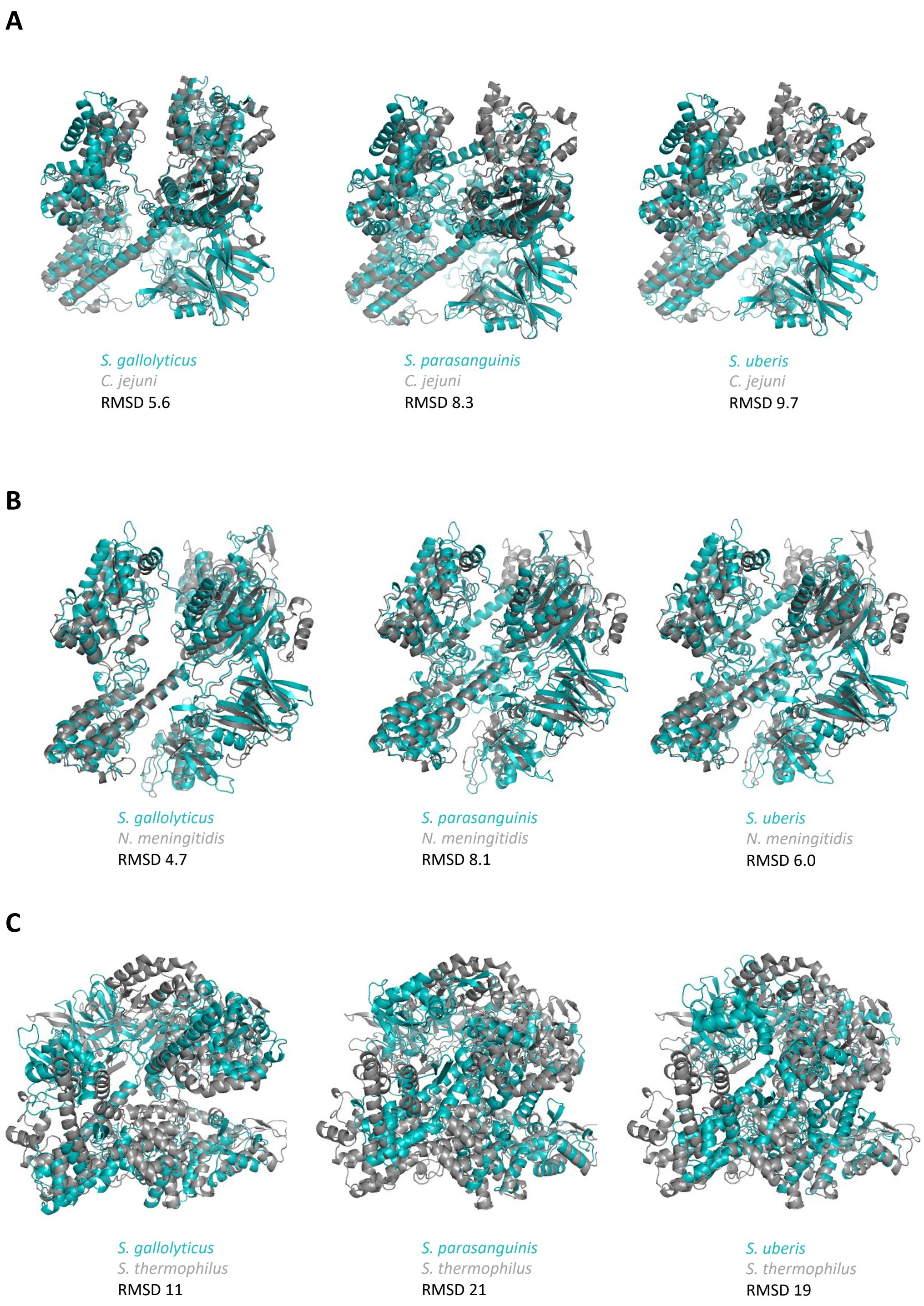


**Supplemental Figure 3.** Structural alignment and RMSD of predicted Cas9 structures for *S. gallolyticus*, *S. parasanguinis*, and *S. uberis* against **(A)** *C. jejuni* **(B)** *N. meningitidis* **(C)** *S. thermophilus.*

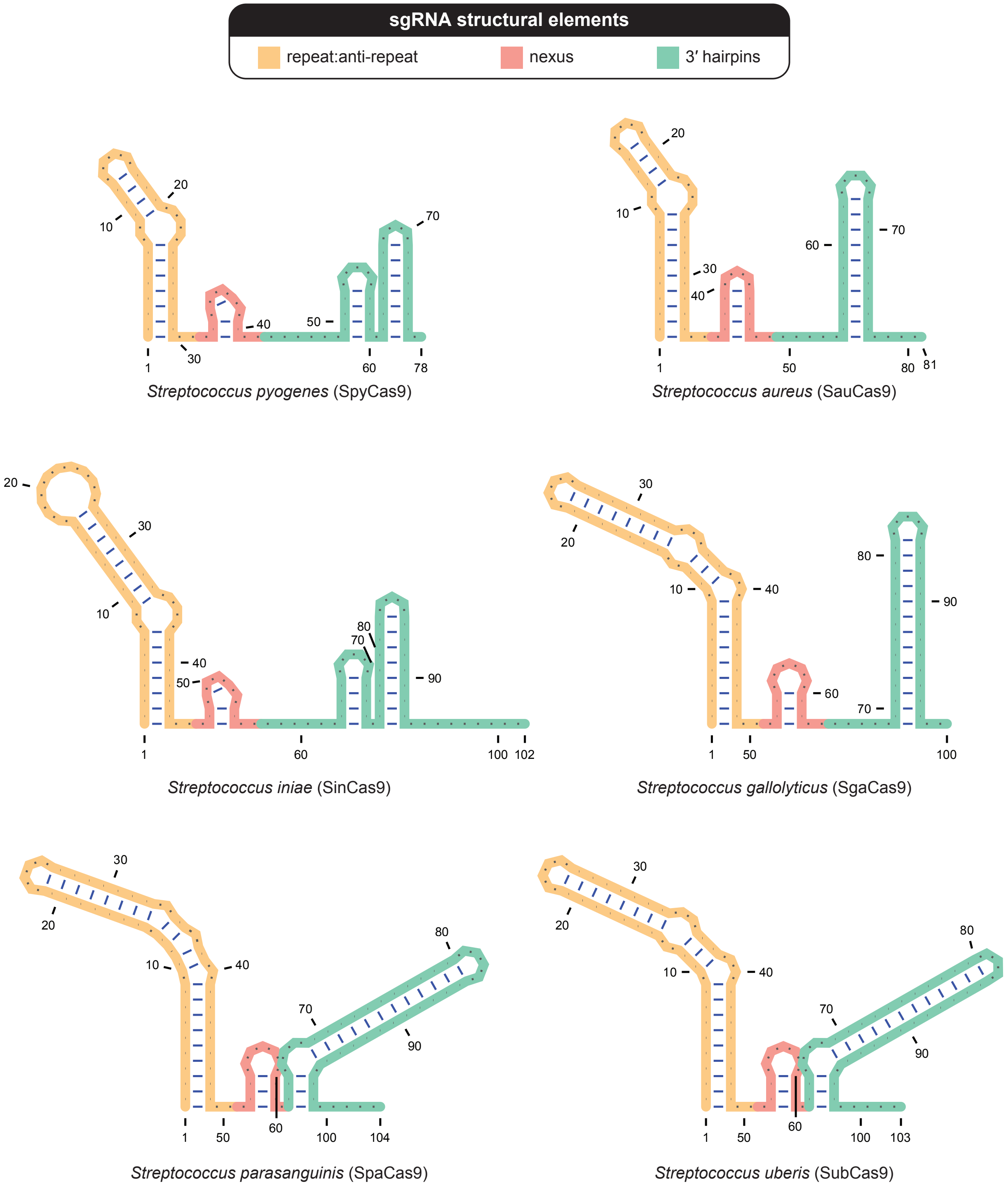


**Supplemental Figure 4.** Predicted sgRNA secondary structures from reference Cas9s Spy and Sau compared with top four orthologous systems in this study: Sin, Sga, Spa, and Sub. Distinct structural elements are indicated by color: the repeat:anti-repeat duplex is highlighted in yellow, nexus in pink, and 3' hairpins in green. Secondary structures of sgRNAs predicted with RNAFold 2.5.1 and visualized with Varna-api 1.2.0[55-56].

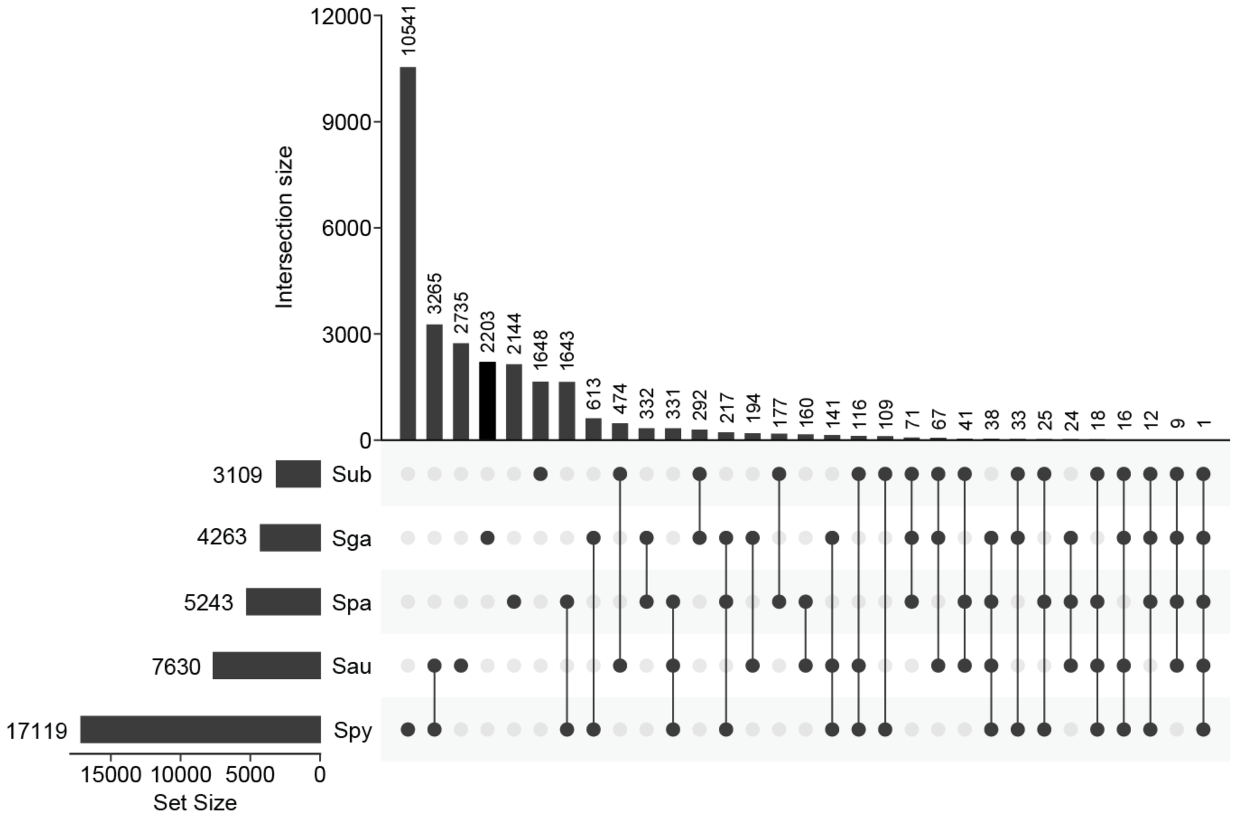


**Supplemental Figure 5**. **Expanded base editing target space provided by Cas9 orthologs.** Upset plot showing the theoretical targetability of ClinVar single nucleotide variants by base editors derived from each Cas9 ortholog. Our newly characterized Cas9 orthologs collectively enable targeting of an additional 6,867 single nucleotide variants not targetable by SauCas9 and Spycas9 base editors, significantly expanding the potential for precision genome editing.

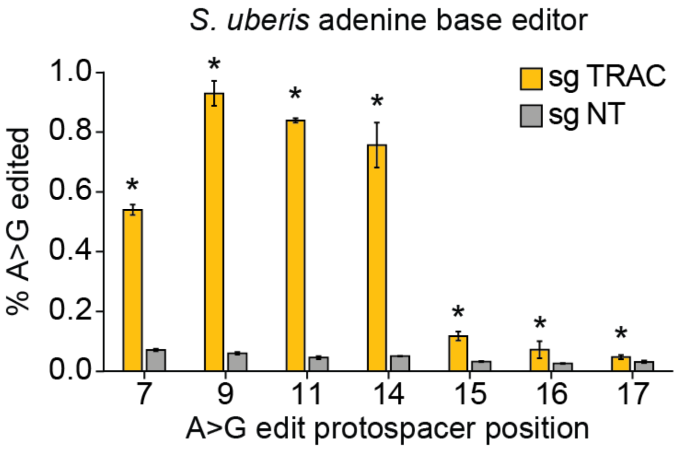


**Supplemental Figure 6**. Adenine base editing activity of *S. uberis* TadA-nCas9. HEK293T cells were transfected with *S. uberis* TadA-nCas9 and sgRNA. Adenine to guanine conversion rates were quantified by deep sequencing. * $p < 0.05$, student's t-test comparing targeting to non-targeting sgRNA.

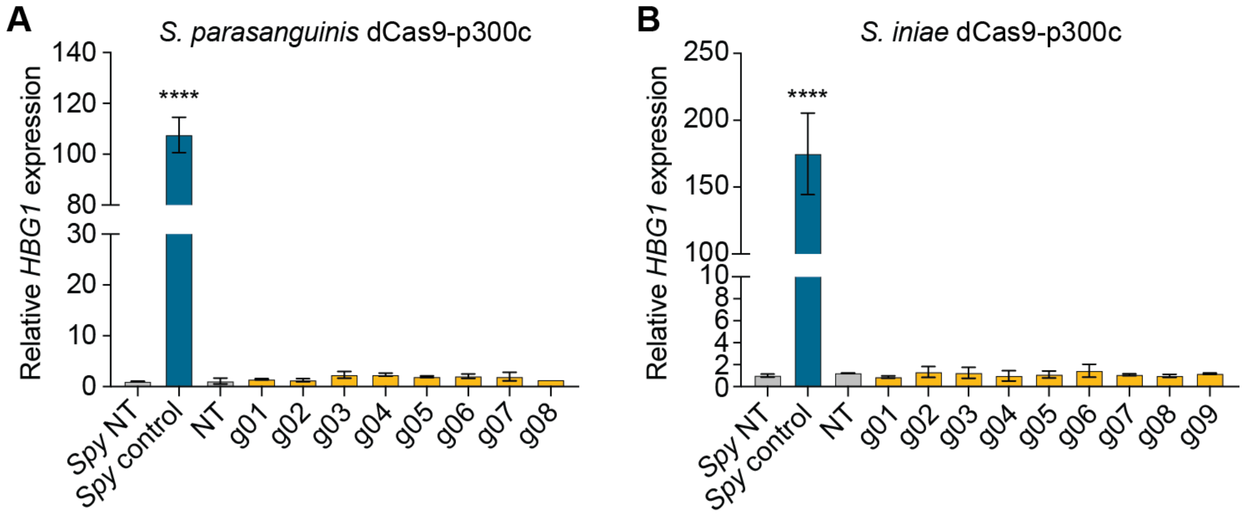


**Supplemental Figure 7. (A-B)** RT-qPCR of *HBG1* expression in HEK293T cells expressing dCas9-p300c from *S. parasanguinis* **(A)** or S. iniae **(B)** dCas9 orthologs and corresponding sgRNAs targeting the *HBG1* promoter. **** $p < 0.0001$, One-way ANOVA on ΔCt values normalized to *GAPDH*, followed by Dunnett's multiple comparison test relative to S. pyogenes Cas9 non-targeting control. multiple comparisons. *n=2* independent biological replicates.
